# The Functional Roles of the *Cis*-acting Elements in *Bamboo mosaic virus* RNA Genome

**DOI:** 10.3389/fmicb.2017.00645

**Published:** 2017-04-13

**Authors:** I-Hsuan Chen, Ying-Wen Huang, Ching-Hsiu Tsai

**Affiliations:** Graduate Institute of Biotechnology, National Chung Hsing UniversityTaichung, Taiwan

**Keywords:** positive-sense RNA virus, *Bamboo mosaic virus*, *cis*-acting elements, viral RNA replication, potexvirus

## Abstract

*Bamboo mosaic virus* (BaMV), which belongs to the genus *Potexvirus* in the family *Alphaflexiviridae*, has a single-stranded positive-sense RNA genome that is approximately 6400 nucleotides (nts) in length. Positive-sense RNA viruses can use genomic RNA as a template for translation and replication after entering a suitable host cell. Furthermore, such viral RNA is recognized by capsid protein for packaging and by viral movement protein(s) or the movement protein complex for cell-to-cell and systemic movement. Hence, viral RNA must contain signals for different functions to complete the viral infection cycle. In this review, we examine various *cis*-acting elements in the genome of BaMV. The highly structured 3′ untranslated region (UTR) of the BaMV genomic RNA plays multiple roles in the BaMV infection cycle, including targeting chloroplasts for RNA replication, providing an initiation site for the synthesis of minus-strand RNA, signaling for polyadenylation, and directing viral long-distance movement. The nt at the extreme 3′ end and the structure of the 3′-terminus of minus-strand RNA are involved in the initiation of plus-strand genomic RNA synthesis. Both these regions have been mapped and reported to interact with the viral-encoded RNA-dependent RNA polymerase. Moreover, the sequences upstream of open reading frames (ORFs) 2, 3, and 5 are involved in regulating subgenomic RNA synthesis. The *cis*-acting elements that were identified in BaMV RNA are discussed and compared with those of other potexviruses.

## Introduction

For a positive-sense RNA virus to establish a successful infection in a host, the viral RNA must house diverse *cis*-acting elements for minus-strand, plus-strand, and possibly subgenomic RNA syntheses (Dreher, 1999; Newburn and White, 2015). Furthermore, *cis*-acting elements could also be involved in cell-to-cell or systemic movement and encapsidation of viral RNA (Kwon et al., 2005; Lough et al., 2006; Cho et al., 2012; Rossmann, 2013). Studying the mechanisms of viral infections, localizing these *cis*-acting elements, and revealing their functional structures are critical steps in understanding viral infections at the molecular level. A few approaches were used to determine the minimum length and structures of viral *cis*-acting elements required for various functions. An *in vitro* replication assay is one of the most frequently used strategies to define the minimal requirement of *cis*-acting RNA elements for replication (Lin et al., 2005a,b; Osman et al., 2014). However, the difficulty involved in isolating a competent replicase preparation that can synthesize minus- or plus-strand RNAs, specifically with the *cis*-acting elements provided, limitsits use. The *cis*-acting elements discovered using this strategy were designated as promoters and are structured to specifically interact with the replicase. Structures of *cis*-acting elements have been computationally predicted and validated using enzymatic or chemical structural probing (Cheng and Tsai, 1999; Sun and Simon, 2006; McCormack et al., 2008). However, the structured *cis*-acting elements must be functionally verified by mutational analysis in either an *in vitro* replication assay or an *in vivo* infection assay.

**FIGURE 1:**
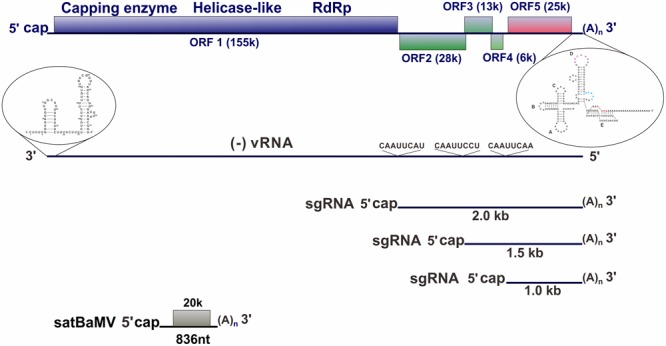
**Illustration of the genome organization of BaMV.** BaMV genome, the minus-strand RNA indicated as (–)vRNA, three subgenomic RNAs (sgRNA), and satellite BaMV (satBaMV) are illustrated. The promoter structures for minus- and plus-strand RNA synthesis at the 3′ UTR of BaMV genome and the 3′-end of (–)vRNA, respectively, are indicated. The conserved sequence among the promoters of BaMV sgRNA synthesis is also indicated on the (–)vRNA.

*Bamboo mosaic virus* (BaMV) has a single-stranded positive-sense RNA genome that is approximately 6.4 kb in length with a 5′-cap structure and a 3′ poly(A) tail. The genome contains five open reading frames (ORFs) (**Figure 1**). ORF1 encodes a replicase for viral RNA replication, ORF2 encodes a 28-kDa protein (a silencing suppressor) required for viral movement, ORF3 and ORF4 encode membrane-anchoring proteins required for virus movement, and ORF5 encodes a 25-kDa capsid protein for viral encapsidation, movement, and symptom development.

The *cis*-acting elements of BaMV RNA involved in viral RNA replication, intracellular trafficking, and movement have been extensively studied in the last two decades. This report comprehensively reviews these studies and discusses the common theme of the roles of these *cis*-acting elements that could be applied to other members of potexvirus including the *Potato virus X* (PVX), one of the top 10 plant viruses in molecular plant pathology (Scholthof et al., 2011), and even to certain animal viruses of *Alphaviruses*.

## Viral RNA Intracellular Trafficking

When a positive-sense viral RNA enters a host cell, the host translation system is used to synthesize the viral proteins. The newly translated viral proteins target a specific membrane, usually an organelle-associated membrane, and modify the membrane suitable for viral RNA replication (Ahlquist et al., 2003; Laliberte and Sanfacon, 2010; Diaz et al., 2012; Nagy, 2016). The RNAs of *Tobacco mosaic virus* (Kawakami et al., 2004; Nishikiori et al., 2006), PVX (Bamunusinghe et al., 2009), *Tomato ringspot virus* (Han and Sanfacon, 2003), *Cowpea mosaic virus* (Carette et al., 2000), and *Tobacco etch virus* (Schaad et al., 1997) are transported to the endoplasmic reticulum membranes. The RNA of *Tomato bushy stunt virus* is transported to the peroxisomes (McCartney et al., 2005). The RNA of *Melon necrotic spot carmovirus* is associated with the mitochondria (Mochizuki et al., 2009). The RNAs of *Turnip yellow mosaic virus* (Prod’homme et al., 2003) and *Turnip mosaic virus* (Wei et al., 2010) are transported to the chloroplast membranes. These observations indicate that different viruses associate with distinct organellar membranes for replication (Laliberte and Sanfacon, 2010).

The mechanisms underlying the specific trafficking of viral RNA to targeted organelles for replication remain less known. In a recent study, BaMV was demonstrated to associate with chloroplasts for replication (Cheng et al., 2013). When the interaction between the 3′ untranslated region (UTR) of BaMV RNA (**Figure 1**) and host proteins in the replicase complex was studied, the involvement of elongation factor 1a (eEF1a) and chloroplast phosphoglycerate kinase (PGK) was revealed (Lin et al., 2007). A further study of the interactions indicated that a pseudoknot, including the poly(A) sequence at the extreme 3′ end, is the target of PGK. *In vitro* and *in vivo* studies revealed that the interaction is required for efficient replication (Lin et al., 2007; Cheng et al., 2013). Notably, the chloroplast PGK can be replaced by a chimeric protein composed of cytoplasmic eEF1a and chloroplast RuBisCo small subunit (rbcS) (Cheng et al., 2013). These results suggest that nuclear-encoded chloroplast proteins, such as PGK and rbcS may serve to transport chloroplast-unrelated macromolecules into the chloroplasts by using their transit peptide. Once inside the host cell, the 3′-terminal pseudoknot and poly(A) sequence of BaMV RNA interact with PGK. The chloroplast PGK transit peptide facilitates entry into the chloroplast transport system. PGK and its associated macromolecules (BaMV RNA and possibly the translated replicase or the entire replicase complex) are transported into the chloroplasts (Cheng et al., 2013).

## Minus-Strand RNA Synthesis

During initiation of minus-strand RNA synthesis, *cis*-acting elements located at the 3′ end (usually in the 3′ UTR) in most viruses play a critical role in recognition by the replicase complex. Typically, the 3′-terminal nucleotides (nt) or penultimate nt of non-poly(A)-tailed RNA viruses is used as the initiation site for minus-strand RNA synthesis (Dreher, 1999, 2009). However, the poly(A)-tailed RNA viruses have RNA genomes containing approximately 250 adenylates at the 3′ end in the case of BaMV (Chen et al., 2005). Thus, *cis*-acting elements in the 3′ UTR are far from the extreme 3′ end of the initiation site if poly(A)-tailed viruses use a similar synthesis mechanism as described for non-poly(A)-tailed RNA viruses. Therefore, poly(A)-tailed RNA viruses might have a different strategy or use initiation sites that are close to the *cis*-acting elements. *In vitro* and *in vivo* studies of BaMV revealed that the extreme 5′ end of minus-strand RNA contains stretch of uridine residues ranging from 1 to 15 nt, usually about 7–10 uridines (Cheng et al., 2002). These results indicate that the replicase complex assembles on the *cis*-acting elements in the 3′ UTR, and that synthesis of minus-strand RNA initiates with uridylate. The consequence of minus-strand RNA synthesis is that the subsequently synthesized plus-strand genomic RNA would have only a short stretch of adenylates at the extreme 3′ end (<15 nts in length).

*Cis*-acting elements for minus-strand RNA synthesis, as mentioned previously, are usually situated in the 3′ UTR and form secondary or tertiary structures, such as the stem–loops of *Alfalfa mosaic virus* (Houser-Scott et al., 1994; Reusken and Bol, 1996; Houser-Scott et al., 1997) and *Turnip crinkle virus* (Song and Simon, 1995) and the tRNA-like structures of *Brome mosaic virus, Tobacco mosaic virus*, and *Turnip yellow mosaic virus* (Kao and Sun, 1996; Osman and Buck, 1996; Deiman et al., 1997, 1998; Singh and Dreher, 1997).

**FIGURE 2:**
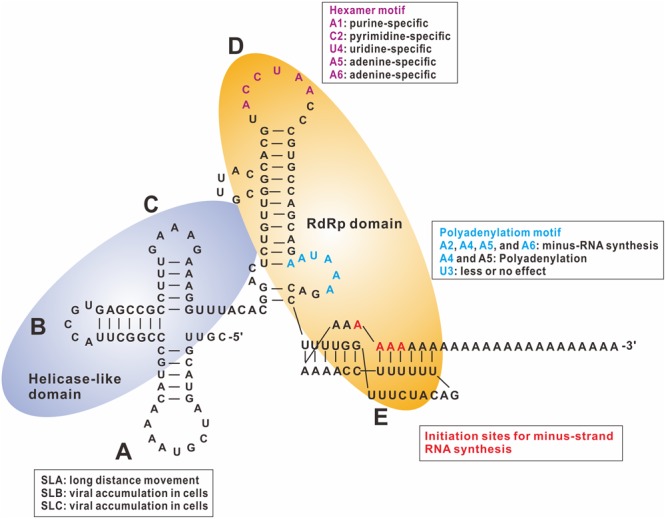
**Tertiary structure of BaMV 3′ UTR and replicase binding sites.** The tertiary structure of BaMV 3′ UTR interacts with the RNA-dependent RNA polymerase (RdRp) and the helicase-like domains shown in light orange and light purple ovals, respectively. The functional roles of these *cis*-acting elements in the 3′ UTR are indicated in boxes. SL: stem–loop (adapted from Cheng et al., 2007).

The *cis*-acting elements for BaMV minus-strand RNA synthesis were also identified in the 3′ UTR. The 3′ UTR can be divided into three portions: the 5′ part consisting of three stem–loops that form a cloverleaf-like structure, designated as the ABC domain; the middle part following the ABC domain, which is a major stem–loop with a bulge and an internal loop, designated as the D domain; and the 3′ part of the UTR that forms the pseudoknot, described previously that interacts with eEF1a and PGK, covering a part of the poly(A) sequence adjoining the 3′ UTR, designated as the E domain (**Figure 2**). Furthermore, results derived from ultraviolet (UV)-crosslinking and foot-printing assays indicate that the polymerase and helicase-like domains of the replicase (ORF1 of BaMV) interact with the D and E domains and ABC domain of the 3′ UTR, respectively. The potexviral conserved hexamer motif (ACXUAA) involved in the accumulation of virus was discovered in *Clover yellow mosaic virus* (White et al., 1992) and BaMV (Cheng and Tsai, 1999). This motif is located at the apical loop of the D domain in the 3′ UTR of BaMV (**Figure 2**), and was protected from RNase digestion during interaction with the polymerase (Huang et al., 2001). The results of mutagenesis of this motif (ACCUAA in BaMV) indicate that the extreme 5′ adenylate is a purine-specific nt, and the subsequent nt is by necessity a pyrimidine. The last three residues (UAA) are unalterable. The third nt affects viral accumulation less than the first two (Chiu et al., 2002).

Maintaining the structures of D and E domains is critical for efficient viral RNA replication (Tsai et al., 1999). Mutations that disrupt the stems resulted in inefficient accumulation of viral RNAs. When compensatory mutations were introduced to re-form the stems, viral replication was restored. Furthermore, retaining the pseudoknot structure of the E domain required 15 adenylates downstream (Cheng and Tsai, 1999). Viral full-length transcripts with <10 adenylates could not replicate sufficiently to be detected in the protoplasts. Transcripts with 15 adenylates at the 3′ end could accumulate only up to 26% of the amount of wild-type transcripts with 25 adenylates (Tsai et al., 1999). These results suggest that the polymerase domain of the BaMV replicase interacts with stem–loop D specifically with the hexamer motif (ACCUAA) and the pseudoknot for initiation of minus-strand RNA synthesis. The initiation site for the minus-strand RNA synthesis in BaMV is not fixed at one position, but initiation starts at one of the 15 adenylates adjoining the 3′ UTR (Cheng et al., 2002).

The stem–loops B and C of the ABC domain in the 3′ UTR play a lesser, but significant, role in RNA replication (Chen et al., 2003). Accumulation of viral RNA in mutants with deleted stem–loop B or C was approximately 30% of wild type. Notably, accumulation of viral products of mutants with deleted stem–loop A did not differ significantly from that of wild type in protoplasts and inoculated leaves, but accumulation decreased dramatically in systemic leaves. These results suggest that stem–loop A is a *cis*-acting element for long-distance movement and does not play a role in RNA replication (**Figure 2**).

## Plus-Strand RNA Synthesis

**FIGURE 3:**
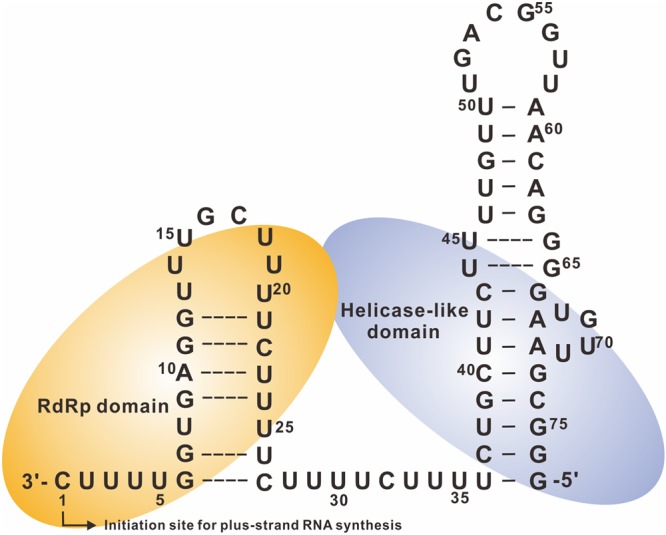
**Secondary structure of the 3′-terminal 77 nts of BaMV minus-strand RNA and replicase binding sites.** The secondary structure of the *cis*-acting elements for genomic RNA synthesis interacts with the polymerase (RdRp) and helicase-like domains shown in light orange and light purple, respectively. The broken lines between base-pairing indicate that this region could be unstructured or has potentially unstable according to the probing results. The initiation site is numbered +1 for plus-strand RNA synthesis (adpated from Cheng et al., 2007).

In an *in vitro* transcription assay, short transcripts of 39, 77, and 173 nts in length, corresponding to the 3′ terminus of minus-strand RNAs, were used as templates to examine their ability to direct RNA synthesis. The 3′-terminal 77-nt RNA, designated Ba-77, was the most efficient RNA template (**Figure 3**). It harbors two complete stem–loops confirmed by enzymatic structural probing and is required for plus-strand RNA synthesis (Lin et al., 2005a).

The terminal UUUUC pentamer is the most critical *cis*-acting element in BaMV for plus-strand RNA synthesis. Ba-77/Δ5, which lacks the terminal pentamer, exhibited only 7% template activity compared with that of Ba-77 *in vitro*. Ba-77 with an internal deletion of 16 or 31 nts (starting after the terminal pentamer) (**Figure 3**) and retaining the terminal UUUUC preserves up to 60% of the template activity of Ba-77 (Lin et al., 2005a). Furthermore, the sequence and structure of the large stem–loop at the extreme 5′ end of Ba-77 are also vital for RNA synthesis. In mutants with altered sequences of the large stem–loop, RNA synthesis *in vitro* and viral RNA accumulation *in vivo* decreased significantly. Moreover, the sequence between the terminal pentamer initiation site and the large stem–loop may also play a significant role as mutants with shortening the sequence between the terminal UUUUC and the stem–loop exhibited decreased accumulation of BaMV RNA *in vivo* and plus-strand RNA synthesis *in vitro* (Lin et al., 2005a).

At least three *cis*-acting elements at the 3′ end of BaMV minus-strand RNA are required for efficient plus-strand genomic RNA synthesis, namely the 3′-terminal UUUUC pentamer motif, the sequence and structure of the large stem–loop, and the distance between these two regions. Accordingly, these*cis*-acting elements constitute the promoter for genomic RNA synthesis. The replicase contains two domains that interact with the promoter: the replicase catalytic center interacts with the terminal UUUUC sequence, and the specificity domain interacts with the large stem–loop (**Figure 3**) (Chen et al., 2010).

As mentioned previously, the extreme 5′ end of minus-strand RNA has a short run of uridylates, copying from the poly(A) tail; therefore, the extreme 3′ end of plus-strand RNA immediately after synthesis accordingly has a short run of adenylates (most frequently 7–10). However, to maintain the approximately 250 adenylates at the extreme 3′ end of genomic RNA after synthesis, the *cis*-acting element AAUAAA in the 3′ UTR plays a role in polyadenylation (Chen et al., 2005). Interestingly, the *cis*-acting element for polyadenlyation of BaMV RNA is identical to that of nuclear-encoded mRNAs. Whether BaMV uses an identical set of proteins as do nuclear-encoded host mRNAs for polyadenylation is an interesting question. A few observations oppose the aforementioned hypothesis on the use of identical proteins for polyadenylation. The host poly(A) polymerase is located mainly in the nucleus. Furthermore, the polyadenylation of mRNAs with poly(A) polymerase is independent of the recognition of the AAUAAA motif, whereas the polyadenylation of BaMV is associated with the AAUAAA motif (Chen et al., 2005).

## Subgenomic RNA Synthesis

The genomes of many positive-sense RNA viruses are multicistronic organizations that produce subgenomic RNAs (sgRNAs) to serve as messengers, allowing the translation of downstream ORFs (Sztuba-Solinska et al., 2011). A few strategies for synthesizing sgRNAs have been demonstrated, including internal initiation (Miller et al., 1985; Haasnoot et al., 2000), premature termination (White, 2002; Jiwan and White, 2011), and discontinuous synthesis (Sawicki and Sawicki, 1998; Pasternak et al., 2001). A short non-coding RNA derived from genomic RNA generated by host exonuclease is another strategy to synthesize subgenomic RNA (Iwakawa et al., 2008). They commonly rely on *cis*-acting RNA elements to direct the viral-encoded RdRp to transcribe these RNAs (Newburn and White, 2015).

**FIGURE 4:**
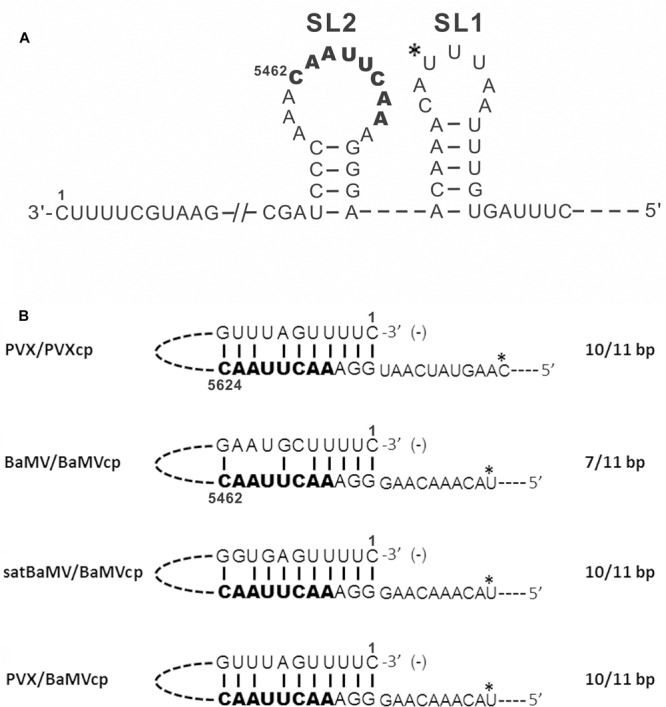
**Putative long-distance RNA–RNA interactions for subgenomic RNA synthesis. (A)** The predicted structure of the core sequence for BaMV 1-kb sgRNA synthesis (adapted from Lee et al., 2000). **(B)** Putative interaction of the 3′-end sequence of PVX, BaMV, or satBaMV minus-strand RNA with the conserved octamer motif upstream of the BaMV or PVX coat protein gene are indicated. The conserved octamer motif is indicated in bold font. The subgenomic RNA transcription start site is indicated using an asterisk. The predicted complementary base pairing is denoted.

BaMV infection produces three sgRNAs with 3′ cotermini. Two major sgRNAs of approximately 2 and 1 kb in length direct translation of ORF2 and ORF5, respectively (Lin et al., 1992). The other sgRNA, responsible for the translation of ORF3 and ORF4, is 1.5 kb in length accumulates in infected cells at a very low level. The satellite RNA of BaMV (satBaMV) was previously designed to be an expression cassette for examining *cis*-acting elements required for sgRNA synthesis (Lee et al., 2000). A cDNA covering the putative promoter region of BaMV sgRNA (SGP) was inserted into this cassette and resulted in sgRNA promoter-directed RNA synthesis in infected cells when coinoculated with BaMV. The *cis*-acting element of the SGP for synthesis of the 1-kb sgRNA covers the region between nt -91 to +52 (the transcription start site is designated as +1). Further analysis indicated that the SGP can be split into four elements: the core (nt -30 to +16), two upstream enhancers (nt -59 to -31 and -91 to -60), and a downstream enhancer (nt +17 to +52). The core sequence is the minimum region required for 1-kb sgRNA synthesis, which folds into two stem–loops, stem–loop (SL)1 and SL2, in minus strand (**Figure 4A**). Maintaining the integrity of SL2 structure and the conserved octamer motif (3′-CAAUUCAA-5′) in the loop are essential for 1-kb sgRNA synthesis. Furthermore, the *cis*-acting elements of SGP for 2-kb sgRNA synthesis are located at nt -119 to +11 (the transcription start site of the 2-kb sgRNA is designated as +1). The minus-strand SGP sequence for 2-kb sgRNA synthesis was predicted to have similar stem–loops to those of the 1-kb SGP. The conserved octamer motif (3′-CAAUUCAU-5′) is also located in the loop of SL2 (Lee et al., 2000). Furthermore, the expected octamer motif (3′-CAAUUCCU-5′) for BaMV 1.5-kb sgRNA is located 12-nt upstream of the transcription start site.

Compared with the putative SGPs of ORF2 and ORF5 of potexviruses, the octamer motif is highly conserved (Kim and Hemenway, 1997; Lee et al., 1998). The long-distance RNA–RNA interaction between the conserved octamer motif and the 3′-terminal sequence of minus-strand genomic RNA was demonstrated to be required for transcription of PVX sgRNAs (**Figure 4B**) (Kim and Hemenway, 1999). As in an ortholog, a similar interaction was revealed in BaMV with shorter complementary pairing than those in PVX (**Figure 4B**). Although BaMV SGPs were inserted into the satBaMV cassette without a BaMV minus-strand 3′-terminal sequence, the octamer motif could also interact with the 3′-terminal sequence of minus-strand satBaMV RNA (**Figure 4B**) (Lee et al., 2000). Redundant SGPs in a PVX-based expression vector were found to lead to genetic instability. The heterologous SGP from BaMV used in the PVX vector improves its stability for long-term production of proteins (Dickmeis et al., 2014). Complementarity between the octamer motif from the BaMV SGP and the 3′-terminal sequence of the minus-strand genomic RNA is required for transcription of sgRNA synthesis (**Figure 4B**). The long-distance RNA–RNA interaction between of the 3′-terminal sequence and the conserved octamer motif of the SGPs observed in PVX and BaMV favors the internal initiation mode of sgRNA synthesis.

## Concluding Remarks and Future Prospectives

For accomplishing an efficient infection by a positive-sense RNA virus, the viral genome consists of various *cis*-acting elements for intracellular trafficking to organellar membranous target sites, minus-strand RNA synthesis, plus-strand genomic RNA synthesis, subgenomic RNA synthesis, viral movements, and viral encapsidation. In this review, we summarize studies of most of the *cis*-acting elements identified in the BaMV genome, except for those involved in viral movement and viral encapsidation. The signal for BaMV genomic RNA encapsidation is very likely in the 5′ UTR, similar to those identified in PVX (Kwon et al., 2005; Karpova et al., 2006; Petrova et al., 2013, 2015). The structural elements and the functional roles for the encapsidation of BaMV RNA will be revealed in the near future.

## Author Contributions

I-HC wrote the section on the plus-strand RNA synthesis. Y-WH wrote the section of subgenomic RNA synthesis. C-HT wrote the rest part of the manuscript.

## Conflict of Interest Statement

The authors declare that the research was conducted in the absence of any commercial or financial relationships that could be construed as a potential conflict of interest.
